# Novel approach to retrograde ureterorenoscopy: a case report of laparoscopic-assisted ureterorenoscopy for minor calyx stone removal

**DOI:** 10.1093/jscr/rjae776

**Published:** 2024-12-12

**Authors:** Daman Tariq, Ayesha Nazeef, Shahzada Nauman Syed, Amit Kumar Thakur

**Affiliations:** Quaid-e-Azam Medical College, Circular Road, Bahawalpur, Punjab 63100, Pakistan; Quaid-e-Azam Medical College, Circular Road, Bahawalpur, Punjab 63100, Pakistan; Department of Nephro-urology, Dialysis and Renal Transplantation, Bahawal Victoria Hospital, Circular Road, Bahawalpur, Punjab 63100, Pakistan; Accident and Emergency Department, Provincial Hospital Malangwa, Balmandir Chowk, Malangwa, Sarlahi 45800, Nepal

**Keywords:** PUJ obstruction, ureterorenoscopy, minor calyx, laparoscopic pyeloplasty

## Abstract

A 16-year-old male presented to OPD with right-sided flank pain associated with lower urinary tract symptoms. CT scan and ultrasound findings demonstrated a 12.9 mm renal stone in the lower calyx and moderate hydronephrosis, respectively. The diagnosis of pelvic ureteric junction obstruction was confirmed by a diethylenetriamine pentaacetate (DTPA) scan. Laparoscopic pyeloplasty was planned but to remove the stone from a hard-to-reach location i.e. minor calyx, ureterorenoscope was introduced from one of the laparoscopic ports. The stone was retrieved successfully. This case represents the implementation of laparoscopic-assisted ureteroscopy instead of conventional retrograde ureteroscopy. This technique warrants further study as it makes the overall procedure more efficient and time-saving.

## Introduction

In a world where kidney issues are on the rise, pelvic-ureteric junction (PUJ) obstruction stands out as the leading cause of congenital hydronephrosis affecting 1 in 500 live births, with a male-to-female ratio of 2:1. It is a condition in which urine flow disruption occurs between kidney pelvis and proximal ureter [1] increasing risk of renal calyceal calculi, presenting a technically challenging situation for urologists [2].

The ‘Anderson–Hynes technique’ of open dismembered pyeloplasty remains the best treatment for PUJ obstruction in the rapidly changing field of medicine today [3]. Nonetheless, revolutionary minimally invasive methods, such as laparoscopic pyeloplasty (LP), are quickly becoming recognized and accepted for treating PUJ obstruction because they have comparable success rates to open surgery and lower patient morbidity [4].

Ureterorenoscopy, a retrograde procedure, can be performed for stones located within the kidney that involves inserting an endoscope into the ureter and calyceal system through the lower urinary tract [2]. There has only been one report of successful one-stage surgery using laparoscopy and ureterorenoscopy to treat bilateral PUJ obstruction with renal stones [5].

Here we present an intriguing case of introducing a ureterorenoscope (URS) through a port during LP, to approach a small stone present in the minor calyx in a patient of the pediatric age group. For the first time in medical literature, a URS was successfully inserted through a port rather than the ureter during LP in a 16-year-old. This successful new time-saving surgical innovation in a pediatric age group patient for the first time in our country with already limited resources makes this case notable and unique.

## Case presentation

**Figure 1:**
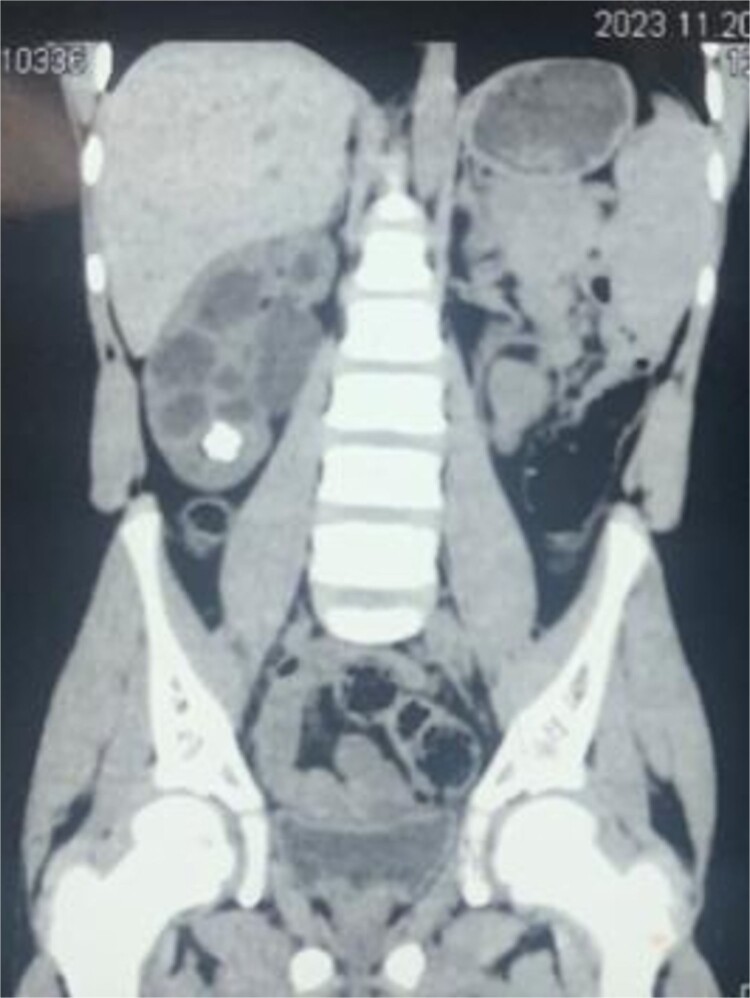
CT scan demonstrating stone in lower pole of right kidney.

**Figure 2:**
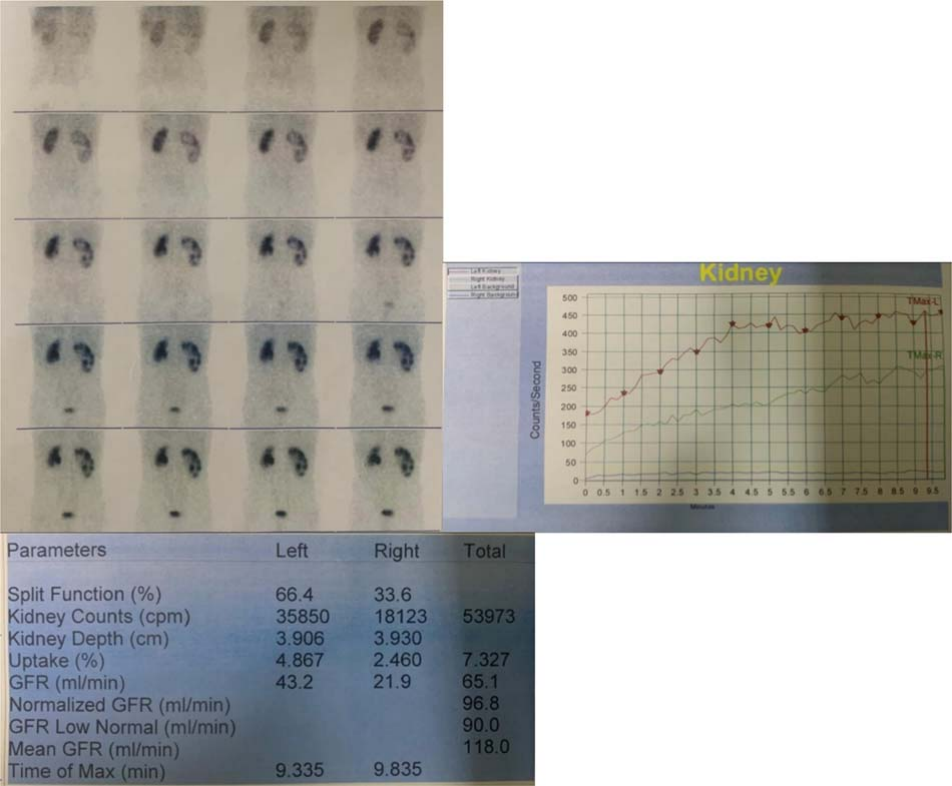
DTPA scan of the patient depicting GFR of right kidney to be 21.9 ml/min.

A 16-year-old male presented into hospital with mainly a complaint of right-sided flank pain that was mild to moderate in intensity, radiating toward the back for a month. The pain was associated with dysuria, urinary urgency and frequency, and occasional hematuria. There was no previous surgical or medical history with insignificant findings on physical examination. His creatinine was 0.5 mg/dl. On ultrasound, there was right renal lithiasis with moderate hydronephrosis, minimal hydroureter, cortical thinning, and pyonephrosis. CT scan demonstrated 12.9 mm calculus in the lower calyx (Fig. 1). Moderate hydronephrosis was visualized with markedly dilated renal pelvis with abrupt narrowing, measuring 38 mm in diameter. On the DTPA renal scan, the glomerular filtration rate (GFR) was 21.9 ml/min (Fig. 2). It showed an obstructive curve which helped in the definitive diagnosis of PUJ obstruction for which LP was planned.

## Surgical procedure

The patient was given general anesthesia along with the administration of IV antibiotics. Foley catheterization was done.

**Figure 3:**
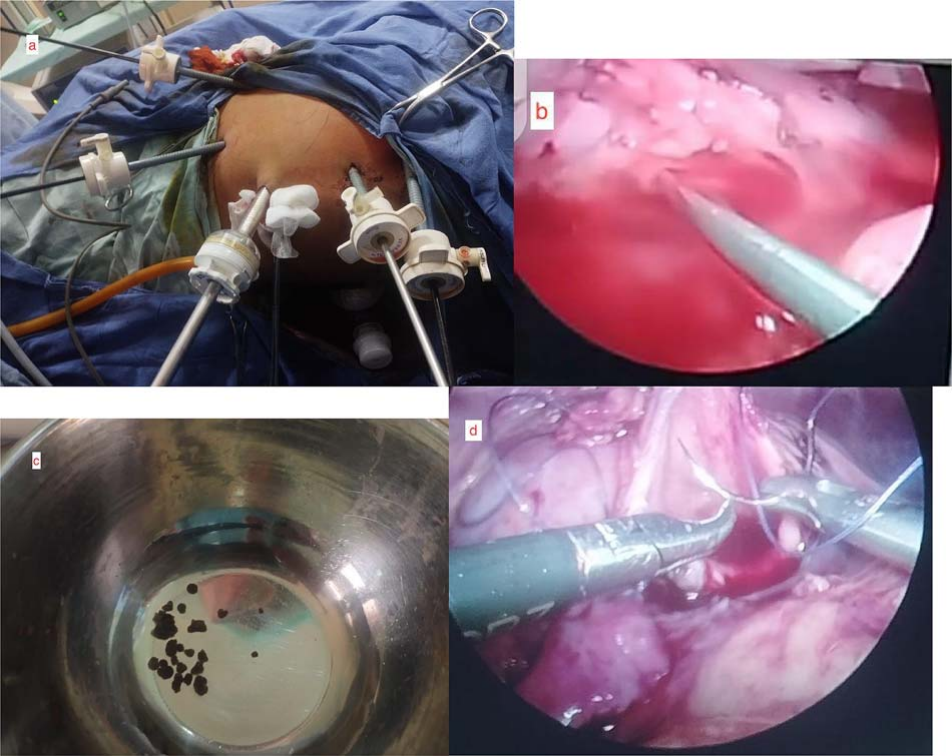
Intraoperative pictures. (a) Assembly of laparoscopic ports. (b) Insertion of URS through the laparoscopic port. (c) Retrieved fragments of 12.9 mm stone from minor calyx. (d) LP repair.

The patient was stabilized in the lateral decubitus position and the pneumoperitoneum was achieved using a veress needle. Two 5 mm ports were placed in the right lower quadrant of the midclavicular line and below the costal margin, respectively, and a 10 mm port was placed 2 cm lateral to the umbilicus. After the Toldt line, the right ureter was identified and traced up to PUJ obstruction medial to the lower pole of the right kidney. The dilated pelvis was mobilized and PUJ obstruction was confirmed. The PUJ obstruction was circumferentially transected and the right ureter was pulled toward the lower pole of the right kidney and spatulated as required. Before forming the anastomosis, renal calculus was detected in the lower pole of the right kidney using a C-arm fluoroscope. The intraoperative decision was made to introduce a URS through one of the 5 mm ports to reach the stone as the stone was present in the minor calyx (Fig. 3).

The stone was approached through URS and intracorporeal pneumatic lithotripsy was done for the fragmentation of the stone and fragments were retrieved through URS forceps. A classic Anderson–Hynes dismembered pyeloplasty was performed using two 4-0 absorbable sutures to complete the anastomosis. A double J stent was placed and the drain was secured.

The blood loss was about 50 ml with an operative time of 2 hours and 30 min. On the fifth postoperative day, the drain was nil and the Foley catheter was removed. Then the patient was discharged uneventfully. After 4 weeks, the double J stent was removed endoscopically. At 3 months follow-up, the patient was symptom-free and showed no signs of obstruction.

## Discussion and conclusion

PUJ obstruction causes abnormal tissue to grow in place of the normal ureter muscle, obstructing urine flow and leading to poor drainage, which adversely affects kidney function due to hydronephrosis, kidney stones, and infections [6, 7]. Open dismembered pyeloplasty, which Anderson and Hynes pioneered for PUJ, has an outstanding success rate and has evolved into the gold standard now [8].

In the last decade, LP has been invented to match open surgery’s success rate with fewer complications and today LP is performed safely in healthcare centers with less morbidity rates than open pyeloplasty [9].

LP is effective for PUJ obstruction patients with renal calyceal stones [10]. These coexisting stones create a therapeutic challenge for urologists. Laparoscopic-assisted dismembered pyeloplasty has shown great success in managing even children, with 40 operations reported [11]. Hence this has proven the success of LP in treating PUJ obstruction with coexisting renal stones as well. By removing and reconstructing the affected area, LP corrects PUJ obstruction. Ureterorenoscopy, another minimally invasive endoscopic procedure, aids in stone diagnosis and precise removal. Kidney stones are removed by retrograde fashion by passing a small telescope (called a URS) up the ureter and through the bladder [2].

A case reporting, laparoscopy and ureterorenoscopy, one-stage surgery, was used to treat PUJ obstruction combined with bilateral kidney stones and the surgical outcomes were ideal [12]. It has been indicated to be both safe and effective in the removal of stones usually present at inaccessible locations like minor calyx using conventional surgical techniques.

The case being presented here is different from the above case as the intraoperative decision was made to introduce a URS through one of the 5 mm ports to reach the stone as the stone was present in the minor calyx. Uretrorenoscopy was not conducted in a normal retrograde fashion but a different approach was used to introduce URS through one of the ports. After that the stone was approached through URS and intracorporeal pneumatic lithotripsy was done for the fragmentation of the stone, the fragments were retrieved through URS forceps.

The purpose of highlighting this innovative surgical method is to report its adaptability and successful results in treating renal stones and PUJ obstruction in adults as well as children. The effectiveness of this groundbreaking approach reveals that it can be used in the future to successfully diagnose and treat renal stones in patients with PUJ obstruction.

The operating time for ureterorenoscopy typically ranges from 20 to 150 min, with a median time of about 60 min [13]. Using a 5 mm port for URS insertion shortens as compared to the time required to introduce a URS through the urethra toward the kidney, confirming its time-saving value. This shows its potential for use in difficult procedures where reducing procedure time is essential, giving physicians a useful alternative to this conventional method.

The technique is less invasive when URS is inserted through a port instead of the urethra, which makes it easier to locate stones in inaccessible areas. These extra advantages of this nontraditional strategy strengthen the case’s originality and convince the reader that it merits more investigation. This new surgical approach, with its ideal outcomes, promises future surgeries as science advances because it makes handling more complex procedures easier and saves valuable time.

According to our knowledge of medical literature, there are very few documented cases of LP and ureterorenoscopy in resource-constrained nations like Pakistan. This is the first instance of a surgical innovation combining these methods that has been successfully applied and produced advantageous outcomes. Amazingly, such results could be attained by a resource-constrained, economically beleaguered country. This breakthrough suggests that with further study, this time-saving, less invasive technique could help doctors in the treatment of numerous patients with PUJ obstruction along with minor calyx calculi, worldwide.
